# MiR-223 plays a protecting role in neutrophilic asthmatic mice through the inhibition of NLRP3 inflammasome

**DOI:** 10.1186/s12931-020-01374-4

**Published:** 2020-05-18

**Authors:** Wenjuan Xu, Yimin Wang, Ying Ma, Jiong Yang

**Affiliations:** 1 Department of Respiratory and Critical Care Medicine, Zhongnan Hospital of Wuhan University, 169 Donghu Road, Wuhan, Hubei 430071 People’s Republic of China; 2 National Clinical Research Center for Respiratory Disease, Guangzhou Institute of Respiratory Health, the First Affiliated Hospital of Guangzhou Medical University, Guangzhou, 510120 China

**Keywords:** miR-223, NLRP3 inflammasome, Airway inflammation, Neutrophilic asthma

## Abstract

**Background:**

Neutrophilic subtype asthma occurs in approximately 15–25% of the asthma cases and is associated with severe airflow obstruction, corticosteroid resistance. MicroRNA plays a vital role in regulating many immune processes, but how miRNA circuits coordinate airway inflammation during neutrophilic asthma is unclear.

**Methods:**

To investigate the molecular mechanism of miR-223 in regulation of neutrophilic airway inflammation, miR-223 knockout mice were used to the OVA/CFA-induced neutrophilic asthma or treated with NLRP3 inhibitor and IL-1β receptor antagonist. Based on the results obtained, wide-type mice were subsequently treated with miR-223 agomirs or negative control agomirs, and the effects on airway inflammation were assessed using morphometric techniques, quantitative RT-PCR, western blot, ELISA and other molecular approaches.

**Results:**

The expression of miR-223 was upregulated in lung tissues of experimental mice model. Furthermore, miR-223^−/−^ mice led to aggravated neutrophilic airway inflammation with heightened histopathological, inflammatory cells and cytokines readouts. Moreover, miR-223^−/−^ mice also presented with enhanced NLRP3 inflammasome level with elevated IL-1β. Blocking NLRP3 or IL-1β diminished this phenotype. Finally, overexpression of miR-223 via treatment with miR-223 agomirs attenuated airway inflammation, NLRP3 levels and IL-1β release.

**Conclusions:**

The findings of this study revealed a crucial role for miR-223 in regulating the immunoinflammatory responses by depressing the NLRP3/ IL-1β axis in neutrophilic asthma.

## Background

Asthma is a heterogeneous chronic disease of the airways characterized by airway inflammation, reversible airway obstruction, and airway hyperresponsiveness (AHR) [1, 2]. Their etiologies are still elusive, because they involve complex interactions between environmental, genetic and immunoregulatory factors. With the progress of the researches, asthma can be divided into eosinophilic asthma (EA) and non-eosinophilic asthma (NEA) according to the presence of granulocytes in the sputum and T-helper cytokine responses [3–6]. A majority of asthmatic patients with eosinophilic inflammation can be well treated by inhaled corticosteroids [7]. However, some with neutrophilic inflammation are often poorly responsive to corticosteroid therapy even at high doses and therefore are at risk of developing refractory asthma [8, 9]. Currently, there are no effective treatments for severe, steroid-resistant and neutrophilic asthma and these patients take up more than half of health care costs [10, 11]. The precise molecular mechanisms leading to neutrophilic inflammation in asthma remain unclear. Thus, understanding the regulatory pathways that control aberrant immune responses in the lung is important to our understanding of neutrophilic asthma pathogenesis.

MiRNAs are small, endogenous, single-stranded, and noncoding RNA molecules that are approximately 18 ~ 22 nt and regulate post-transcriptional gene expression. By complementary base pairing to the 3′ untranslated regions (UTRs) of target genes, microRNAs could lead to mRNA degradation or translation repression of their target genes [12–14]. More recently, the crucial role of miRNA has been implicated in various immunological and inflammatory disorders. Emerging evidences also have revealed the specific miRNA profiles in the development of bronchial asthma, such as miR-16, miR-21, miR-126, miR-145 et al. [15–18]. Recently, miR-223 has been reported to emerge as critical regulators of the response to bacterial stimulation and the immune system [19, 20]. MiR-223 was transcribed from an independent promoter and shown to be specifically expressed in the hematopoietic system [21]. Furthermore, miR-223 played critical roles in the inflammatory diseases by regulating different gene transcription factors, including C/EBPa, NOD-like receptor activation, the ubiquitin ligase Roquin, E2F1, and the NF-κB pathway [22–26]. Recent studies have demonstrated that miR-223–3p was upregulated in sputum of severe asthma and was highest in neutrophilic asthma [27]. However, there are lack of mechanistic studies clarifying how miR-223-regulated gene expression shape airway inflammation in neutrophilic asthma.

The Nod-like receptor protein 3 (NLRP3), a member of NLRs family NLRP3s, which consists of three main proteins, including NLRP3 scaffold, regulatory molecule caspase-1 and apoptosis-associated speck-like protein containing a CARD (ASC), has emerged as a crucial regulator of chronic inflammatory disease [28, 29]. It mediated the activation of caspase-1 in response to microbial ligands, and then cleaved and activated pro-interleukin (IL)-1β and pro-IL-18 to active forms and promotes their secretion [28]. Recent studies have suggested that NLRP3 inflammasome signaling was involved in the pathogenesis of asthmatic inflammation. And the upregulation of NLRP3 and IL-1β in sputum correlated with neutrophilic airway inflammation [30, 31]. Although miR-223 has been proven to suppress NLRP3 expression through combining with the 3′ UTR of NLRP3 [25, 32, 33], the role of miR-223 in the regulation of lung NLRP3 during neutrophilic asthma remains unclear.

In this current study, we aimed to determine whether miR-223 played roles in the regulation of airway inflammation and to investigate the underlying molecular mechanisms in neutrophilic asthma. Our data indicated that miR-223 was upregulated in lung tissues of experimental mice model. miR-223 deficient mice led to aggravated airway inflammation and enhanced NLRP3 inflammasome levels with elevated IL-1β. Collectively, we propose that miR-223 acts as a key rheostat that regulates airway inflammation in neutrophilic asthma.

## Methods

### Mice

Wide-type (WT) mice (CD45.1^+^C57BL/6 mice, 6-8 weeks) were obtained from the Center for Animal Experiments of Wuhan University (Wuhan, China), and were used for the experiments 1 week after arrival. CD45.1^+^miR-223^−/−^ mice were purchased from the Jackson Laboratory. All experimental mice were bred in an approved containment facilities with specific pathogen-free food and water under 12 h light/dark cycle. Experiments were approved by the Institutional Animal Ethics Committee of Wuhan University.

### Induction of neutrophilic asthma model

The experimental protocol for neutrophilic asthma was performed as previously reported [34]. Mice were sensitized on day 0 with 20 μg of grade V ovalbumin (OVA, Sigma Aldrich, St. Louis, MO, USA) emulsified in 75 ul CFA (Sigma Aldrich) by intraperitoneal (i.p.) injection. On days 21 and 22, all mice were challenged with aerosols consisting of 1% OVA (grade III). Control mice received phosphate-buffered saline (PBS) only. The highly selective NLRP3 blocker, MCC950 (200 mg/kg dissolved in PBS, i.p.) [35] and IL-1β receptor antagonist, anakinra (50 mg/kg dissolved in PBS, i.p.) [36] were given to the OVA/CFA-sensitized miR-223^−/−^ mice immediately after each challenge, respectively. Control mice were treated with the same volume of PBS for comparison. Mice were sacrificed 24 hours after the final OVA challenge, and then serum, bronchoalveolar lavage fluid (BALF), lungs were collected for subsequent analysis.

### Agomirs

MiR-223 agomir is a chemically modified oligonucleotide that can be widely used to upregulate the endogenous expression of miR-223 in animal experiments. Agomirs for miR-223 and the negative control were ordered from RiboBio (Guangzhou, China). The sequence of miR-223 agomir were not provided by RiboBio. miR-223 agomirs (5 nmol in 50ul saline) and negative control agomir were administered intranasally on days 20, 21 and 22 [37, 38]. Control mice were treated with the same volume of saline for comparison.

### Bronchoalveolar lavage

The tracheas and lungs were lavaged 3 times via a syringe with 0.5 ml PBS containing 0.6 mM EDTA, as previously described [39]. The BALF was centrifuged at 1500 rpm for 7 min at 4 °C and the BALF supernatant was stored at − 80 °C for cytokine analysis. The recovered BALF cells were prepared by cytocentrifugation (TXD3 cytocentrifuge, Xiangyi, Changsha, China) and were stained with Wright-Giemsa (Jiangcheng Bioengineering Institute, Nanjing, China) for differential cell counts (neutrophils, eosinophils, lymphocytes). Four hundred cells were counted for each slide.

### Lung histopathology

The left lung lobe of each animal was resected and fixed in 4% paraformaldehyde buffer for at least 24 h, then dehydrated and embedded in paraffin. Lung sections were cut into 5-um thickness, and were stained with haematoxylin and eosin (H&E) and periodic acid-Schiff (PAS) to assess airway inflammation, goblet cell hyperplasia and mucus secretion at 200× magnification by microscope. Four sections were assessed per lung.

And a scale was used to semi-quantitatively evaluated the severity of peribronchial and perivascular inflammation, as previously described [40]. The extent of mucus production and goblet cell hyperplasia in the airway epithelium was assessed by calculating Apas+/Pbm using Image Pro Plus 6.0 (IPP 6.0) software [41].

### AHR measurement

Mice were anesthetized with 1% pentobarbital and mechanically ventilated, and AHR was measured by using the animal lung function instrument (Buxco Electronics, Troy, NY, USA), as previously described [42]. Briefly, incremental concentrations of methacholine (ranging from 3.125 to 50 mg/ml) were intratracheally delivered by an attached nebulizer. Baseline airway resistance was assessed using nebulized PBS. Total lung and airway resistance index (RI) were then calculated by the instrument.

### Quantitative RT-PCR

Total RNA was isolated from lung tissue using TRIzol (Invitrogen/Thermo Fisher Scientific, Inc., Carlsbad, CA, USA). Complementary DNA (cDNA) synthesis was performed with a miRNA specific primer using Thermo Scientific RevertAid First Strand cDNA Synthesis Kit according to the manufacturer’s manual. Amplification was performed using qPCR with SYBR Premix Ex TaqTM (Takara Bio Inc., Otsu, Japan). All primers were provided by Sangon Biotech (Shanghai, China), and the primers sequences of target genes are presented in the Table 1. The cycle threshold (Ct) of miRNAs were normalized to the Ct of endogenous U6, whereas GAPDH was used to normalize the expression levels of mRNA. The relative gene expression was calculated by the 2^-ΔΔCq^ method.

**Table 1:** Primer sequences

Gene name	Forward primer	Reverse primer
miR-223	GCGCGTGTCAGTTTGTCAAAT	AGTGCAGGGTCCGAGGTATT
U6	CTCGCTTCGGCAGCACA	AACGCTTCACGAATTTGCGT
NLRP3	GACCAGCCAGAGGTGGAATGA	CTGCGTGTAGCGACTGTTGA
ASC	CACCAGCCAAGACAAGATGA	CTCCAGGTCCATCACCAAGT
Caspase-1	AACAGAACAAAGAAGATGGCACA	CCAACCCTCGGAGAAAGAT
IL-1β	AGTTGACGGACCCCAAAAG	CTTCTCCACAGCCACAATGA
IL-18	TGGAGACCTGGAATCAGACA	TGGGGTTCACTGGCACTT
GAPDH	TGTGTCCGTCGTGGATCTGA	TTGCTGTTGAAGTCGCAGGAG

### Western blot

To measure the proteins expression of NLRP3, ASC, Caspase-1, IL-1β and IL-18, lung tissues were infiltrated in tissue protein regent with RIPA Lysis Buffer and protease inhibitor (Beyotime Institute of Biotechnology, Haimen, China). The protein concentrations were measured using BCA Protein Assay kit (Thermo Fisher Scientific) following the protocol. The total proteins were separated by 10% SDS-PAGE and then transferred onto a PVDF membrane (Millipore Corp., Billerica, MA, USA). The membranes were blocked with 5% non-fat milk in TBST solution for 2 h at room temperature. The PVDF membranes were subsequently incubated with primary antibodies against NLRP3, ASC, Caspase-1, IL-1β and IL-18 (Abcam, Cambreidge, UK) at 4 °C overnight. After three times washes in TBST for 15 min each, the membranes were incubated with HRP-conjugated secondary antibodies at 37 °C for 2 h. Immunoreactive images were captured with an enhanced chemiluminescence kit according to the manufacturers’ protocol and were detected using the ChemiDocTM Imaging System (Bio-Rad). GAPDH was used as an internal reference.

### Cytokine analysis

The levels of IL-4, IL-5, IL-13, interferon gamma (IFN-γ), IL-17A, IL-22, IL-23, IL-1β and IL-18 in BALF were measured using enzyme-linked immunosorbent assay (ELISA) kit (eBioscience, San Diego, CA, USA) according to the manufacturers’ protocols.

### Dual-luciferase reporter assay

To determine the target relationship between miR-223 and NLRP3 mRNA, a luciferase reporter assay was performed using 239 T cells co-transfected with a NLRP3–3’UTR fusion vector and miR-223 mimic, inhibitor and corresponding negative control. Cells were harvested after 48 h, and the luciferase levels were detected using a Dual-Luciferase Reporter Assay System (Promega) according to the manufacturers’ instructions.

### Statistical analysis

Data analyses were performed with Student’s t-test or Analysis of Variance (ANOVA) using SPSS 17.0 software (SPSS; IBM, Armonk, NY, USA). Data were presented as means ± standard deviation (SD). *P* < 0.05 was regarded as statistical significance. All experimental data were repeated at least three times.

## Results

### Increased miR-223 expression in mice with OVA-induced neutrophilic asthma

**Fig. 1:**
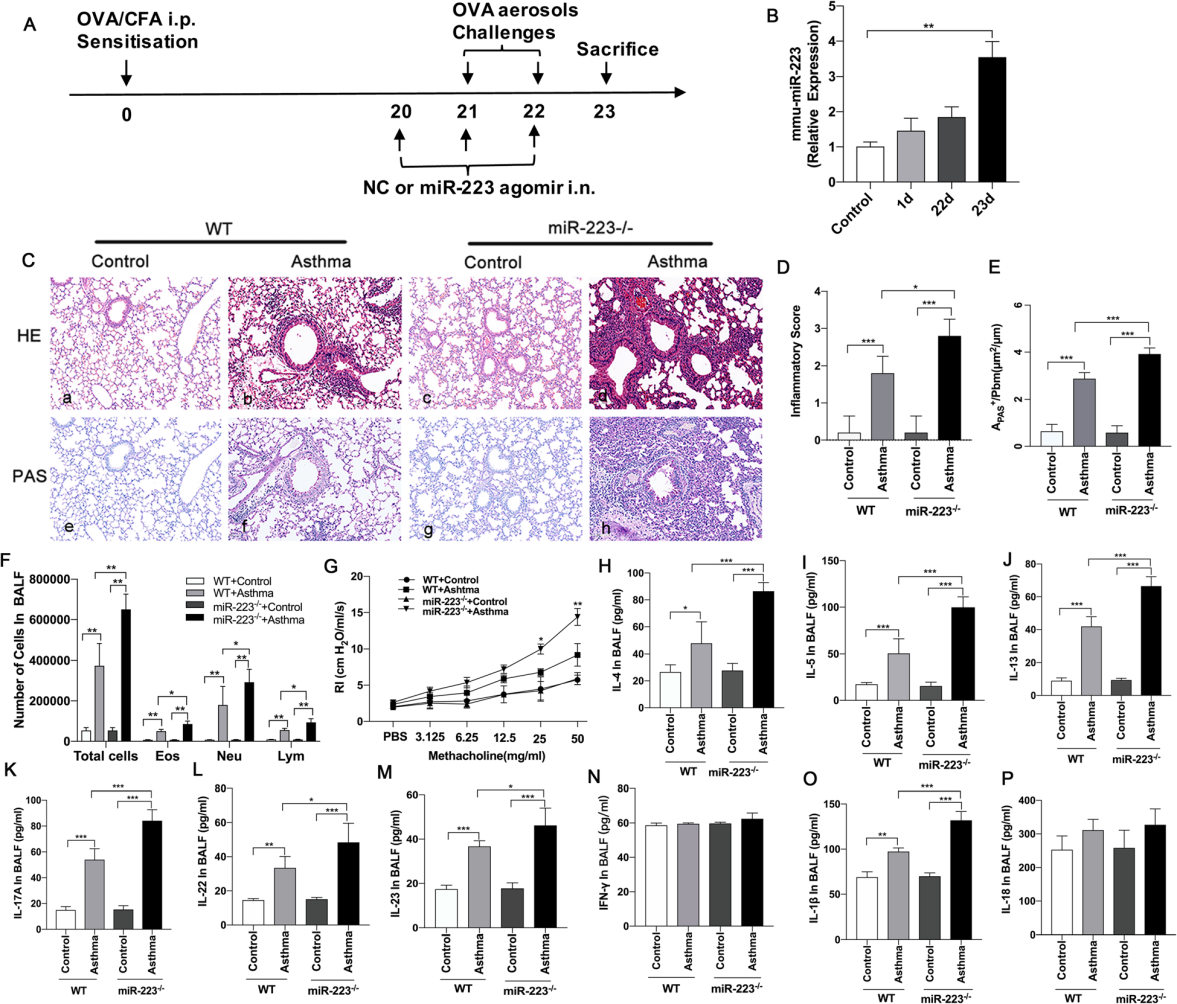
MiR-223 played an important role in regulating neutrophilic airway inflammation in the OVA/CFA-induced asthma model. **a** Mice were sensitized against OVA in the presence of complete Freund’s adjuvant (CFA), followed by exposure to 1% OVA aerosols on days 21 and 22. Administration of miR-223 agomirs or negative control agomirs to OVA-sensitized mice for 3 consecutive days was shown. Analysis was performed at day 23. **b** MiR-223 expression levels were detected in the lung tissues from mice after OVA sensitization (1 day), after first challenged (22 day) and in asthma model (23 day) by qPCR, respectively. *n* = 6–8 mice/group; statistical significance was determined by ANOVA. **c** Representative micrographs of lung H&E staining **a**-**d** and PAS staining **e-h** from different groups (200× magnification). **d, e** Semi-quantification of lung inflammatory score and Apas^+^/Pbm was performed. *n* = 6–8 mice/group; statistical significance was determined by ANOVA. **f** Number of total inflammatory cells, neutrophils, eosinophils, and lymphocytes was calculated in BALF. *n* = 6–8 mice/group; statistical significance was determined by ANOVA. **g** AHR was determined by lung resistance. n = 6–8 mice/group. Statistical significance was determined by ANOVA. **h**-**p** The levels of Th2-associated cytokines (IL-4, IL-5, IL-13), Th17-associated cytokines (IL-17A, IL-22, IL-23), Th1-associated cytokine (IFN-γ), IL-1β, and IL-18 and in BALF were measured by ELISA. *n* = 6–8 mice/group; statistical significance was determined by ANOVA. All data were expressed as mean ± SD. *P < 0.05, **P < 0.01, ***P < 0.001

To investigate the role of specific miRNAs in the OVA-induced neutrophilic asthmatic mice model (Fig. 1.a), we identified that the miR-223 expression level in the lungs of the OVA-induced mice model (23 day) was significantly upregulated compared with the PBS –induced mice (Fig. 1.b). In addition, whereas the expression of miR-223 was increased after sensitization (1 day) and after the first challenge (22 day), there was no significant difference compared with the control group (Fig. 1.b). These evidences provided a basis to assess the biological function of miR-223 during neutrophilic asthma.

### Exacerbated airway inflammation in miR-223^−/−^ mice during neutrophilic asthma

To further elucidate the role of miR-223 in the regulation of neutrophilic inflammation in asthma, we used miR-223^−/−^ mice sensitized and exposed to OVA, followed nebulizing 1%OVA for 2 consecutive days after 3 weeks. MiR-223^−/−^ mice exposed to OVA had dramatically increased numbers of infiltrating inflammatory cells and mucus hypersecretion, as determined by the number of PAS staining cells in airways, when compared with OVA-challenged WT mice (Fig. 1.c-e). No inflammatory cells and PAS-positive cells were found in either WT mice or miR-223^−/−^ mice that were exposed to PBS. Furthermore, miR-223^−/−^ mice exposed to OVA had markedly increased the number of total inflammatory cells, neutrophils, eosinophils and lymphocytes in BALF when compared to OVA-challenged WT mice (Fig. 1.f). In addition, the airway resistance of mice to increasing concentrations of methacholine was detected. The results demonstrated that RI was significantly difference at higher concentrations of methacholine in OVA-challenged miR-223^−/−^ mice compared with OVA-challenged WT mice (Fig. 1.g). In comparison with the OVA-challenged WT mice, OVA-challenged miR-223^−/−^ mice showed a significant reduction in the compliance of lung at the concentration of 3.125 and 25 mg/ml. Subsequently, the expression of Th1-related cytokines (IFN-γ), Th2-related cytokines (IL-4, IL-5, IL-13) and Th17-related cytokines (IL-17A, IL-22, IL-23) in BALF were examined by ELISA, respectively. IL-4, IL-5, IL-13, IL-17A, IL-22, IL-23 levels were markedly raised in BALF of OVA-challenged miR-223^−/−^ mice compared with OVA-challenged WT mice (Fig. 1.h-m). However, IFN-γ release was not significantly altered in OVA-challenged miR-223^−/−^ mice compared with OVA-challenged WT mice (Fig. 1. n). Other cytokines including IL-1β, IL-18 were also detected in BALF. Notably, IL-1β release dramatically increased in miR-223^−/−^ mice compared with OVA-challenged WT mice (Fig. 1. o). Similarly, the expression of IL-18 was also increased in OVA-induced miR-223^−/−^ mice, but the difference was not significant as IL-1β (Fig. 1. P). Thus, these results reveal that miR-223 is associated with airway inflammation, mucus production, AHR, the Th2 and Th17 responses and pro-inflammatory cytokines release in the lung tissues of neutrophilic asthmatic mice.

### Increased NLRP3 inflammasome activity in miR-223^−/−^ mice during neutrophilic asthma

**Fig. 2:**
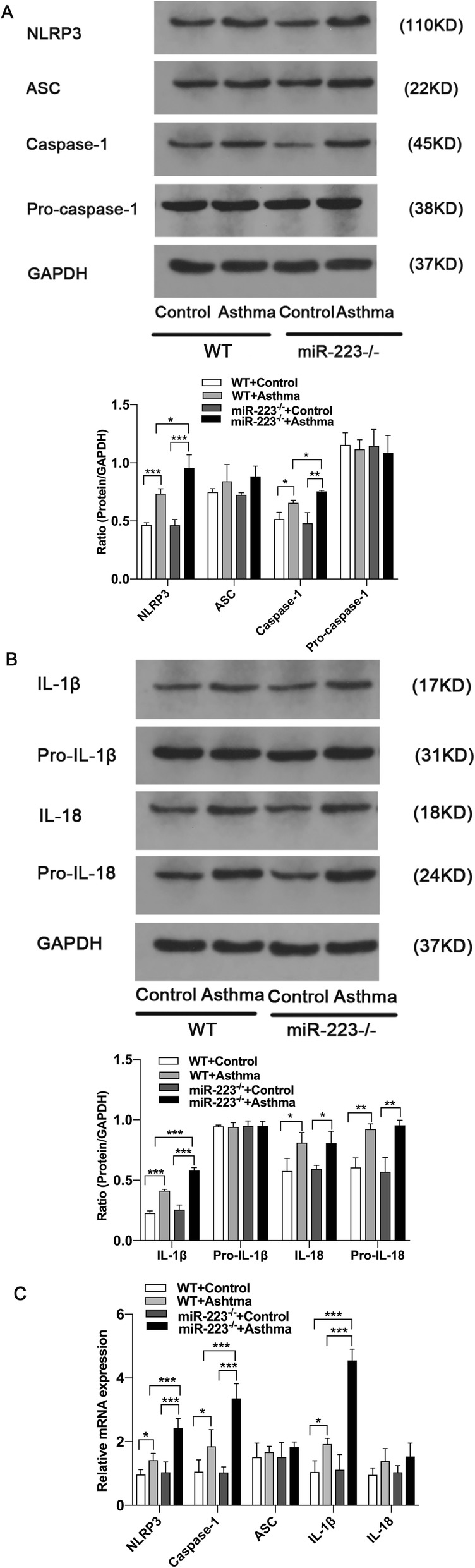
Increased NLRP3 inflammasome activity in miR-223^−/−^ mice during neutrophilic asthma. **a** Proteins expression of NLRP3, ASC, caspase-1, pro-caspase-1 and GAPDH were measured by western blot. Representative immunoblot from three independent mice. **b** Proteins expression of IL-1β, pro-IL-1β, IL-18, pro-IL-18 and GAPDH were measured by western blot. Representative immunoblot from three independent mice. *n* = 6–8 mice/group; statistical significance was determined by ANOVA. **c** Genes expression of NLRP3, ASC, caspase-1, IL-1β and IL-18 were determined by qPCR. n = 6–8 mice/group; statistical significance was determined by ANOVA. All data were expressed as mean ± SD. *P < 0.05, **P < 0.01, ***P < 0.001

Based on the finding that miR-223 regulates airway inflammation and strengthen IL-1β release, we next explored the role for miR-223 target mRNA, NLRP3. To further confirm the effects of miR-223 deficiency on NLRP3 inflammasome, we detected the protein expression of NLRP3 in lung tissues by western blotting. Moreover, the pulmonary expression levels of NLRP3 protein were significantly increased in OVA-challenged miR-223^−/−^ mice compared with OVA-challenged WT mice (Fig. 2.a). Consistent with this result, the expression levels of pro-caspase-1 (45KD) and cleaved caspase-1 (20KD) and IL-1β and IL-18 were also significantly increased. However, miR-223 deficiency did not alter the protein expression of ASC, pro-IL-1β, pro-IL-18 (Fig. 2.a-b). In accordance with these results above, we observed the mRNA expression of NLRP3, Caspase-1, IL-1β were markedly higher in lung tissue of OVA-challenged miR-223^−/−^ mice compared with OVA-challenged WT mice (Fig. 2.c). These data indicate that miR-223 plays a selective and functional role in regulating a critical component of NLRP3 inflammasome.

### Blockade of NLRP3 inflammasome or IL-1β abrogates the enhanced inflammation of miR-223^−/−^ mice during neutrophilic asthma

**Fig. 3:**
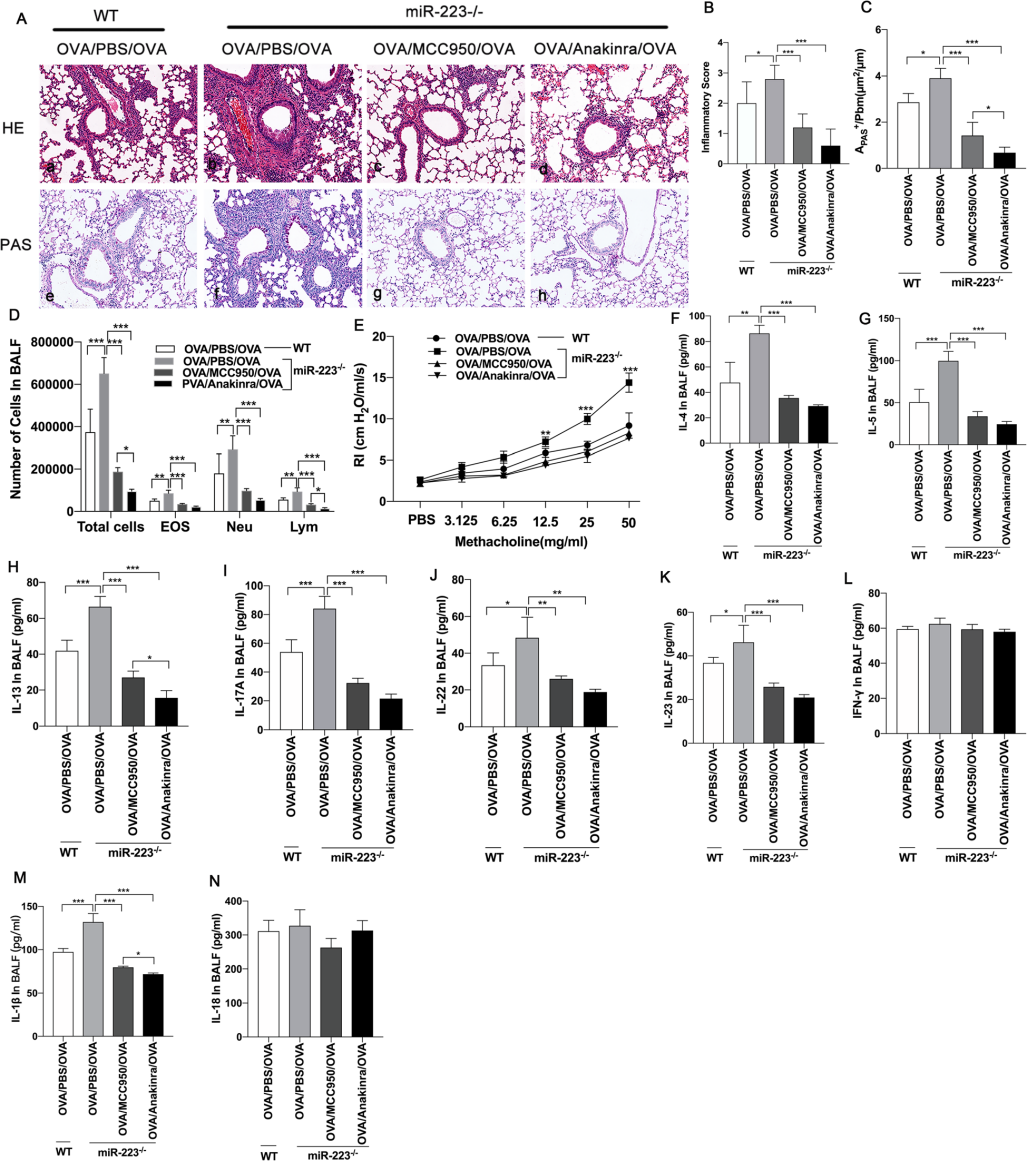
Blockade of NLRP3 inflammasome or IL-1β abrogates airway inflammation in miR-223^−/−^ mice. OVA/CFA was administrated to WT mice and miR-223^−/−^ mice on day 0. miR-223^−/−^ mice received the inhibitor of NLRP3, MCC950 (200 mg/kg, i.p.) and IL-1β receptor antagonist, anakinra (50 mg/kg, i.p.) or PBS after each challenge, respectively. Mice were euthanized 24 h after the final treatment. **a** Representative micrographs of lung H&E staining **a**-**d** and PAS staining **e**-**h** from different groups after treatment (200× magnification). **b**-**c** Semi-quantification of lung inflammatory score and Apas^+^/Pbm was performed. *n* = 6–8 mice/group; statistical significance was determined by ANOVA. **d** Number of total inflammatory cells, neutrophils, eosinophils, and lymphocytes was calculated in BALF after treatment. *n* = 6–8 mice/group; statistical significance was determined by ANOVA. **e** AHR was determined by lung resistance after treatment. *n* = 6–8 mice/group. Statistical significance was determined by ANOVA. **f**-**n** The levels of Th2-associated cytokines (IL-4, IL-5, IL-13), Th17-associated cytokines (IL-17A, IL-22, IL-23), Th1-associated cytokine (IFN-γ), IL-1β, and IL-18 in BALF were measured by ELISA after treatment. n = 6–8 mice/group; statistical significance was determined by ANOVA. All data were expressed as mean ± SD. *P < 0.05, **P < 0.01, ***P < 0.001

**Fig. 4:**
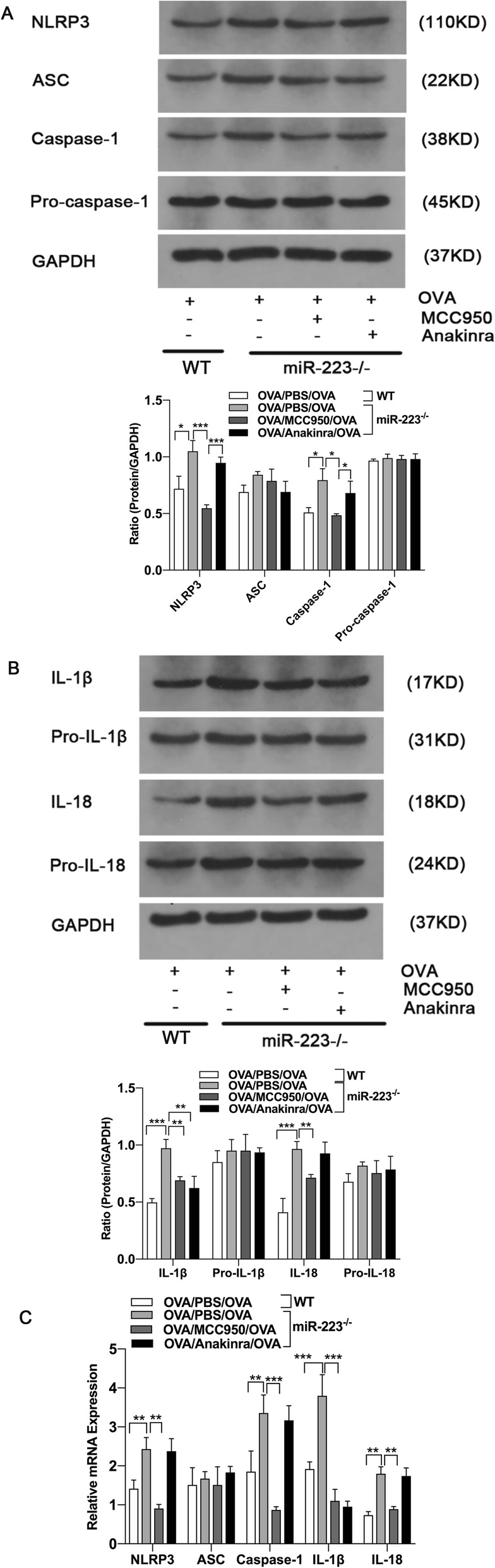
MCC950 suppressed the activation of NLRP3 and reduced pulmonary expression of caspase-1, IL-1β and IL-18 in miR-223^−/−^ mice. **a** Proteins expression of NLRP3, ASC, caspase-1, pro-caspase-1 and GAPDH were measured by western blot after treatment. Representative immunoblot from three independent mice. **b** Proteins expression of IL-1β, pro-IL-1β, IL-18, pro-IL-18 and GAPDH were measured by western blot after treatment. Representative immunoblot from three independent mice. n = 6–8 mice/group; statistical significance was determined by ANOVA. **c** Genes expression of NLRP3, ASC, caspase-1, IL-1β and IL-18 were determined by quantitative PCR after treatment. *n* = 6–8 mice/group; statistical significance was determined by ANOVA. All data were expressed as mean ± SD. *P < 0.05, **P < 0.01, ***P < 0.001

Next we verified whether blocked either the NLRP3 inflammasome or IL-1β could directly further attenuate airway inflammation in miR-223^−/−^ mice. On days 21 and 22 after sensitization, miR-223^−/−^ mice were treated with NLRP3 inhibitor (MCC950) or IL-1 receptor antagonist (anakinra) immediately after each OVA challenge, respectively. Both treatment dramatically decreased the numbers of infiltrating inflammatory cells and mucus hypersecretion in the lung, as well as the number of total inflammatory cells, neutrophils, eosinophils and lymphocytes in BALF (Fig. 3.a-d). Similarly, airway resistances were significantly abolished by both MCC950 and anakinra administration (Fig. 3.e). Although inflammatory cytokines IFN-γ was unaltered, the levels of IL-4, IL-5, IL-13, IL-17A, IL-22, IL-23, IL-1β and IL-18 were significantly lower after treatment (Fig. 3.f-n). To confirm the role of MCC950, the protein levels of NLRP3 and its important components, ASC and caspase-1 and the typical downstream proteins, IL-1β and IL-18 were detected by western blotting. As shown in Fig. 4.a-c, MCC950 administration nearly abolished the protein expression of caspase-1, although it did not alter the protein expression of ASC. Consistent with this, the protein expression of IL-1β was remarkably decreased by treatment with MCC950, and IL-18 also decreased after treatment. On the other hand, the protein expression of pro-IL-1β, pro-IL-18 were unaltered. Thus, enhanced NLRP3 activation followed by mainly increased mature IL-1β resulted in an enhanced airway inflammation in miR-223^−/−^ mice.

### NLRP3 is identified as a direct target of miR-223

To further verify the interaction between NLRP3 and miR-223, bioinformatics analysis was performed to predict the presence of binding sites of miR-223 and the NLRP3 3′-UTR. Similar to previous publications [23], complementary sequences between miR-223 and the NLRP3 3′-UTR was also observed. And dual luciferase reporter assay also showed that the overexpression of miR-223 diminished the luciferase activity of the NLRP3 3’UTR of the wild-type, while the effect was abolished with the mutant NLRP3 3′-UTR. Conversely, inhibition of miR-223 enhanced the luciferase activity with the NLRP3 3’UTR of the wild-type, but not that of the mutant NLRP3 3′-UTR (unpublished data). Collectively, these findings confirmed that NLRP3 was a direct target of miR-223.

### MiR-223 agomirs attenuates airway inflammation in neutrophilic asthma

**Fig. 5:**
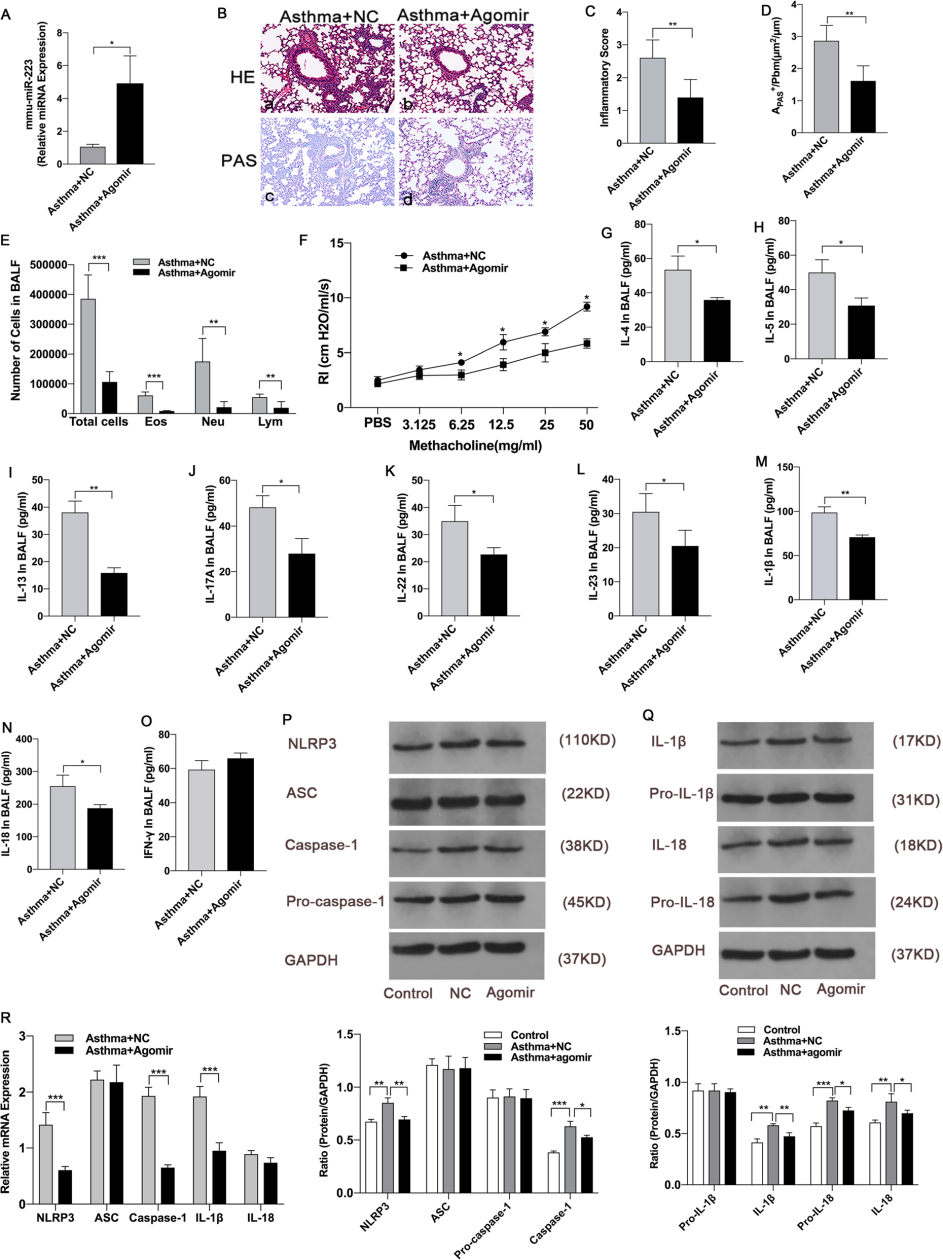
MiR-223 agomirs attenuated airway inflammation in neutrophilic asthma. OVA/CFA was administrated to WT mice on day 0. Mice were treated with 5 nmol miR-223 agomirs or negative control agomirs on days 21–23 before each challenge, respectively. **a** The expression of miR-223 was detected in lung tissues by qPCR. *n* = 6–8 mice/group; statistical significance was determined by unpaired Student’s *t* test. **b** Representative micrographs of lung H&E staining **a**-**b** and PAS staining **c**-**d** from treated mice (200× magnification). **c**-**d** Semi-quantification of lung inflammatory score and Apas^+^/Pbm was performed. *n* = 6–8 mice/group; statistical significance was determined by unpaired Student’s *t* test. **e** Number of total inflammatory cells, neutrophils, eosinophils, and lymphocytes was calculated in BALF after treatment. *n* = 6–8 mice/group; statistical significance was determined by unpaired Student’s *t* test. **f** AHR was determined by lung resistance after treatment. *n* = 6–8 mice/group. Statistical significance was determined by unpaired Student’s *t* test. **g**-**o** The levels of Th2-associated cytokines (IL-4, IL-5, IL-13), Th17-associated cytokines (IL-17A, IL-22, IL-23), Th1-associated cytokine (IFN-γ), IL-1β, and IL-18 in BALF were measured by ELISA after treatment. *n* = 6–8 mice/group; statistical significance was determined by unpaired Student’s *t* test. **p** Proteins expression of NLRP3, ASC, caspase-1, pro-caspase-1 and GAPDH were measured by western blot after treatment. Representative immunoblot from three independent mice. **q** Proteins expression of IL-1β, pro-IL-1β, IL-18, pro-IL-18, and GAPDH were measured using western blotting after treatment. Representative immunoblot from three independent mice. *n* = 6–8 mice/group; statistical significance was determined using ANOVA. **r** Genes expression of NLRP3, ASC, caspase-1, IL-1β and IL-18 were determined by qPCR after treatment. *n* = 6–8 mice/group; statistical significance was determined by unpaired Student’s *t* test. All data were expressed as mean ± SD. *P < 0.05, **P < 0.01

To explore the effect of miR-223 on airway inflammation in vivo, miR-223 agomirs was delivered intranasally to mice 6 h before OVA-challenge (Fig. 1.a). As shown in Fig. 5.a, the expression of miR-223 in lungs after transfection agomirs was increased fivefold compared with the negative control. Notably, treatment with miR-223 agomirs significantly resulted in a reduction of infiltrating inflammatory cells and mucus hypersecretion in lungs compared with the negative control group (Fig. 5.b-d). In comparison with the negative control group, the inflammatory cells including neutrophils, eosinophils and lymphocytes in BALF were remarkably reduced in miR-223 agomirs treatment group (Fig. 5.e). Besides, remarkable differences in the airway resistances were observed between miR-223 agomirs treatment group and negative control group (Fig. 5.f). Compared with negative control group, miR-223 agomirs treatment effectively reduced the expression of IL-4, IL-5, IL-13, IL-17A, IL-22, IL-23, IL-1β and IL-18 in BALF, while there was no difference in IFN-γ expression between the two groups (Fig. 5.g-o). Furthermore, miR-223 agomirs treatment inhibited the expression of NLRP3 at the level of mRNA and the protein. This was accompanied with the repression of caspase-1and IL-1β protein and mRNA expression (Fig. 5.p-r). Overall, these results confirm that miR-223 play a critical role in regulating airway inflammation and represent a basic study for the application of miRNA in the treatment of asthma.

## Discussion

Although the importance of miRNAs in the regulation of immunological processes has been recognized [43–45], its specific role in the pathogenesis of neutrophilic asthma remains unclear. In this study, we demonstrated that miR-223 participated in the regulation of neutrophilic airway inflammation in the asthma model. MiR-223 expression was upregulated in the lungs of OVA-induced WT mice compared with PBS-induced WT mice, and miR-223^−/−^ mice exposure to OVA resulted in aggravation airway inflammation, mucus hypersecretion and the production of Th2 and Th17 cytokines. In addition, OVA-induced miR-223^−/−^ mice exacerbated AHR, another important feature of asthma [46]. Moreover, both NLRP3/caspase-1 and IL-1β levels were higher in the lungs of OVA-induced miR-223^−/−^ mice compared with those in PBS-induced WT mice. Intranasal administration of miR-223 agomirs not only partially restored airway inflammation, mucus hypersecretion, AHR, the production of Th2 and Th17 cytokines, but also decreased the expression levels of NLRP3/caspase-1 and IL-1β releases. Collectively, these findings suggested that miR-223 played a crucial role in regulation of neutrophilic airway inflammation, and involved in the pathogenesis of neutrophilic asthma.

MiRNAs, small non-coding RNA molecules, have been identified in the development and responses of the immunological and inflammatory disorders. It has been described that unique miRNA expression profiles participate in different phenotypes of asthma [27]. Previous studies have showed that miR-223–3p, miR-142-3p, and miR-629-3p expression were increased in the sputum of neutrophilic asthmatic patients [27]. Similarly, altered miR-223 expression in the bronchial epithelial brushings of patients with mild asthma was reported by Solber et al. [47]. MiR-223 was reported to be emerged as a negative regulator of neutrophils activation in many experimental models of inflammatory diseases. Neutrophils were anticipated to be closely related to the underlying pathophysiology of severe asthma [48–51]. An earlier study by Johnnidis et al. addressed that miR-233 played a regulatory role on neutrophil function [21], which indicated that miR-223 might exert effects on the neutrophilic asthma. Inconsistent with the above results, another study showed that miR-223 expression in asthma was showed to be down-regulated [52] or no alternation in bronchial biopsy specimens of patients with mild asthma, severe asthma and healthy controls [53, 54]. Thus, whether miR-223 participates in the pathogenesis of neutrophilic asthma is still unclear. In this present study, we performed experiment mice model to investigate the potential role of miR-223 in neutrophilic asthma. Our results demonstrated that miR-223 expression was higher in the lungs of neutrophilic asthma mice compared with those in control mice. This finding was in accordance with prior studies. Moreover, we found the deletion of miR-223 aggravated airway inflammation in the OVA-induced neutrophilic asthma mice model. It may be that when asthma occurs, protective factors and harmful factors played roles at the same time. And the occurrence of asthmatic airway inflammation was the result of counterbalance between favorable factors and harmful factors. miR-223 might play a protective role in asthma, so airway inflammation was exacerbated after miR-223 was knocked out. Therefore, these findings showed that miR-223 may be involved in the pathogenesis of neutrophilic asthma.

IL-1β, a potent inflammatory cytokine, was involved in multiple chronic inflammatory diseases, including chronic obstructive pulmonary disease (COPD) and asthma. Recent studies have showed that overexpression of IL-1β and IL-18 might play central roles in the pathogenesis of neutrophilic asthma [31, 55–58]. Inhibition of IL-1β activity by administration of neutralizing antibody or deletion of the IL-1 receptor type I abrogated the progression of asthma, and administration of recombinant IL-1β replicated the markers of neutrophilic asthmatic inflammation [59]. IL-18 knockout mice exhibited decreased neutrophilic inflammation and airway remodeling in OVA-induced asthma [60]. In this present study, we found that the expression of IL-1β and IL-18 in BALF were significantly upregulated in neutrophilic asthma group compared with those in control group, which were consist with the findings of other recent studies [61, 62], implying that IL-1β and IL-18 participated in the pathogenesis of neutrophilic asthmatic inflammation.

Caspase-1, an endogenous cysteine protease and the effector of inflammasome, was required for the cleavage and activation of pro-IL-1β and pro-IL-18, which was involved in inflammation. As caspase-1 activating platforms, inflammasome played a central role in multiple inflammatory diseases, including neutrophilic asthma [28, 58]. Among them, NLRP3 inflammasome was the most fully characterized that dominated the main auto-activation of caspase-1. Kim et al. showed that blockade of NLRP3 in steroid-resistant murine asthma potently inhibited the neutrophilic airway inflammation and AHR, suggesting that NLRP3/caspase-1 played central roles in the pathogenesis of refractory asthma [30, 59]. In this present study, we detected the protein levels and mRNA expression of NLRP3 and caspase-1 in the lung tissues of neutrophilic asthma. Our findings showed that the expression of NLRP3 and caspase-1 increased in the lungs of neutrophilic asthma group compared with those in control group, which were consist with the results of other studies [30, 35]. Administration of MCC950, a highly specific small-molecule inhibitor of NLRP3 inflammasome, significantly suppressed NLRP3 expression levels and caspase-1 activity, alongside with the reduction of IL-1β and IL-18 release, resulted in the diminution of neutrophilic airway inflammation and hyperresponsiveness, indicating that NLRP3/caspase-1/IL-1βsignaling axis was involved in the pathogenesis of neutrophilic asthma.

NLRP3 inflammasome-dependent, IL-1β-mediated IL-17 responses have been associated with neutrophilic airway inflammation and AHR [63, 64]. OVA/CFA-induced asthma was characterized by a large number of neutrophils infiltration in the airways representing Th17-dominant responses and weaker TH2 responses. In the present study, we found that treatment with MCC950 significantly inhibited both Th2 (such as IL-4, IL-5, IL-13) and Th17 (such as IL-17A, IL-22, IL-23) responses in neutrophilic asthma, and reduced the infiltration of neutrophils and eosinophils into the airway and AHR. Anyway, treatment with MCC950 exerted similar effects to IL-1 antagonist [65], implying that NLRP3/ caspase-1/ IL-1β axis was involved in both Th2 and Th17 responses induced by OVA. This was in agreement with other studies showing that blockade of NLRP3 inhibited both eosinophilic and neutrophilic inflammation in severe asthma [30, 59]. Collectively, these evidences suggested that inhibition of NLRP3/ caspase-1/IL-1β axis diminished neutrophilic airway inflammation through restraining Th2 and Th17 responses.

Agomir, a chemically modified oligonucleotide, has been widely used to upregulate the endogenous expression of miRNAs in vivo [66]. Previous studies found that NLRP3 inflammasome activity was negatively controlled by miR-223, which played a role in inflammatory diseases [23, 25, 67, 68]. In this present study, miR-223 overexpression with agomirs attenuated airway inflammation, AHR and pro-inflammatory cytokines production. MiR-223 agomirs could effectively suppress the mRNA and protein expression of NLRP3, but miR-223 deficiency largely promoted the mRNA and protein expression of NLRP3. Blockade of NLRP3 resulted in significant repression of airway inflammation and pro-inflammatory cytokines production, mimicking the biological effects of miR-223 overexpression. Dual-luciferase reporter assay was performed to verify the interaction between NLRP3 and miR-223 as previously descripted [23], the data demonstrated that miR-223 directly targeted on the 3’UTR of NLRP3 mRNA. In light of these finds, it will be interesting to reveal that miR-223 regulates the neutrophilic airway inflammation by directly regulating the expression of NLRP3 and may be a potential target for the treatment of neutrophilic asthma in the future.

## Conclusions

In summary, the present study has demonstrated that the miR-223-NLRP3/IL-1β regulatory circuit plays a protective role in neutrophilic airway inflammation. Upregulation miR-223 can inhibit the airway inflammation and pro-inflammatory cytokines production through inhibiting the activation of NLRP3/IL-1β signaling pathway in mice, which is involved in the pathogenesis of asthma. Collectively, this study may provide a promising target for the treatment of neutrophilic asthma in the future.

## Data Availability

The dataset supporting the conclusions of this article is included within the article.

## Abbreviations

OVA: Ovalbumin
PBS: Phosphate-Buffered Saline
BALF: Bronchoalveolar Lavage Fluid
H&E: Haematoxylin and Eosin
PAS: Periodic Acid-Schiff
AHR: Airway Hyperresponsiveness
EA: Eosinophilic Asthma
NEA: Non-eosinophilic Asthma
UTRs: Untranslated Regions
NLRP3: Nod-like Receptor Protein 3
ASC: Apoptosis-associated Speck-like Protein Containing a CARD
IL-1β: Interleukin-1β
EDTA: Ethylenediaminetetraacetic Acid
RI: Resistance Index
Cdyn: Dynamic Compliance
cDNA: Complementary DNA
Ct: Cycle Threshold
PVDF: Polyvinylidene Fluoride
TBST: Tris-buffer-saline and Tween20
IFN-γ: Interferon Gamma
ELISA: Enzyme-linked Immunosorbent Assay
ANOVA: Analysis of Variance
SD: Standard Deviation
